# Primary Bilateral Adrenal Lymphoma Revealed by Adrenal Insufficiency: From Constitutional Symptoms to Crisis

**DOI:** 10.7759/cureus.113562

**Published:** 2026-07-28

**Authors:** Keiko Carter, Abanoub Khalil, Mohamad Abufaied, Ravikumar Ravindran

**Affiliations:** 1 Internal Medicine, University Hospital of Wales, Cardiff, GBR; 2 Internal Medicine, Royal Gwent Hospital, Aneurin Bevan University Health Board, Newport, GBR; 3 Diabetes and Endocrinology, Cwm Taf Morgannwg University Health Board, Cardiff, GBR; 4 Diabetes and Endocrinology, Aneurin Bevan University Health Board, Newport, GBR

**Keywords:** adrenal crisis, adrenal insufficiency, diffuse large b‑cell lymphoma, non-hodgkin’s lymphomas, primary bilateral adrenal lymphoma

## Abstract

Primary adrenal lymphoma (PAL) is an uncommon extranodal non‑Hodgkin lymphoma that typically presents with bilateral adrenal masses and carries a poor prognosis, especially when complicated by adrenal insufficiency; primary bilateral adrenal lymphoma is a rare but important cause of adrenal insufficiency and adrenal crisis, particularly when large bilateral adrenal masses are detected on imaging.

A 50‑year‑old man presented with several months of constitutional symptoms, including night sweats, vomiting, weight loss, diarrhoea and myalgia, and was initially treated conservatively with symptom resolution, but four months later he was readmitted with anorexia, further weight loss, fever and left upper quadrant pain; imaging revealed bilateral adrenal masses (6.7 cm right, 4.5 cm left) with high non‑contrast attenuation (>20 Hounsfield units) and splenomegaly, and MRI confirmed progression of the right adrenal lesion to 8.5 cm. Biopsy demonstrated diffuse large B‑cell lymphoma consistent with PAL, and he was commenced on hydrocortisone and fludrocortisone replacement together with eight cycles of R‑CHOP (rituximab, cyclophosphamide, doxorubicin hydrochloride (hydroxydaunorubicin), vincristine sulfate (Oncovin), and prednisolone) and intrathecal methotrexate, achieving complete radiological remission sustained to 2016, with remission maintained until his death from sepsis and multi‑organ failure in 2023.

This case illustrates the broad symptom spectrum of PAL, from B-symptoms to adrenal crisis, and underscores the importance of considering PAL in the differential diagnosis of bilateral adrenal masses with high attenuation. Early recognition of adrenal insufficiency and adrenal biopsy are critical to guide timely initiation of life-saving glucocorticoid replacement and lymphoma-directed therapy.

## Introduction

Primary adrenal lymphoma (PAL) is a rare entity, accounting for <1% of non‑Hodgkin lymphomas and a small fraction of adrenal malignancies. It typically presents in older men with bilateral adrenal involvement in up to 75-80% of reported cases, and adrenal insufficiency is documented in approximately 50-70% of patients [1-5]. Patients usually present with non-specific constitutional or abdominal symptoms, including fever, weight loss, fatigue, abdominal pain and occasionally adrenal crisis, which contributes to frequent diagnostic delay and misclassification as metastatic disease, infection, pheochromocytoma or adrenocortical carcinoma [4,5].

Radiologically, PAL often appears as large bilateral adrenal masses with high unenhanced CT attenuation, low contrast washout and heterogeneous enhancement, features that favour malignancy but are not pathognomonic [1,6]. Histologically, diffuse large B-cell lymphoma is the predominant subtype, and overall prognosis has historically been poor, with reported median survival around 12-18 months in many series [1]. However, outcomes have improved in the rituximab era, and case reports and series show that early recognition, prompt endocrine stabilisation, and timely lymphoma-directed chemotherapy can occasionally achieve complete and even long-term remission, as illustrated by the present patient [1,7].

Early diagnosis is clinically important because untreated adrenal insufficiency can rapidly progress to life-threatening adrenal crisis, while delay in biopsy or chemotherapy may allow further progression of an aggressive but potentially treatment-responsive lymphoma [4,6]. Management is also challenging because clinicians must simultaneously exclude pheochromocytoma, secure tissue diagnosis, start steroid replacement when adrenal failure is suspected, and coordinate systemic therapy for a rare high-risk extranodal lymphoma [4]. This case adds to the literature by illustrating a diagnostically challenging presentation of PAL with adrenal insufficiency, documenting a detailed endocrine and haematological work-up, and demonstrating durable remission despite the usually guarded prognosis of this condition [1,7].

## Case presentation

A 50‑year‑old man with no known prior endocrine disease presented in April 2015 with a six‑month history of night sweats, vomiting, progressive weight loss (12 kg over 3 weeks), loose stools, poor appetite, cough with white sputum and diffuse myalgia. CT showed splenomegaly and inflammatory mesenteric changes, without clear evidence of malignancy. Initial assessment suggested a systemic inflammatory or infective process; his symptoms improved with supportive management, and he was discharged at the end of May 2015.

**Figure 1:**
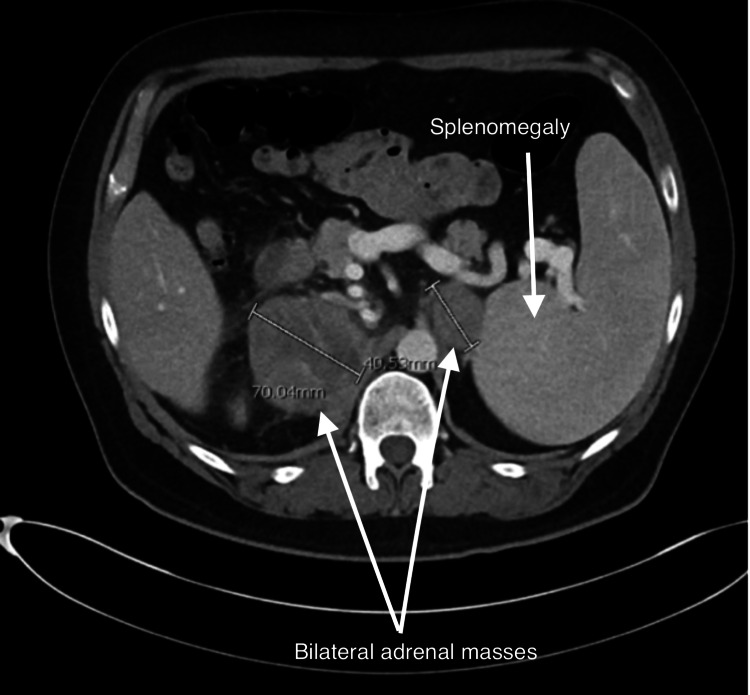
CT abdomen and pelvis with contrast Bilateral adrenal masses (6.7 cm right, 4.5 cm left), indistinct from adrenal glands. Splenomegaly (18 cm), no focal splenic lesion, with high Hounsfield units >20.

**Figure 2:**
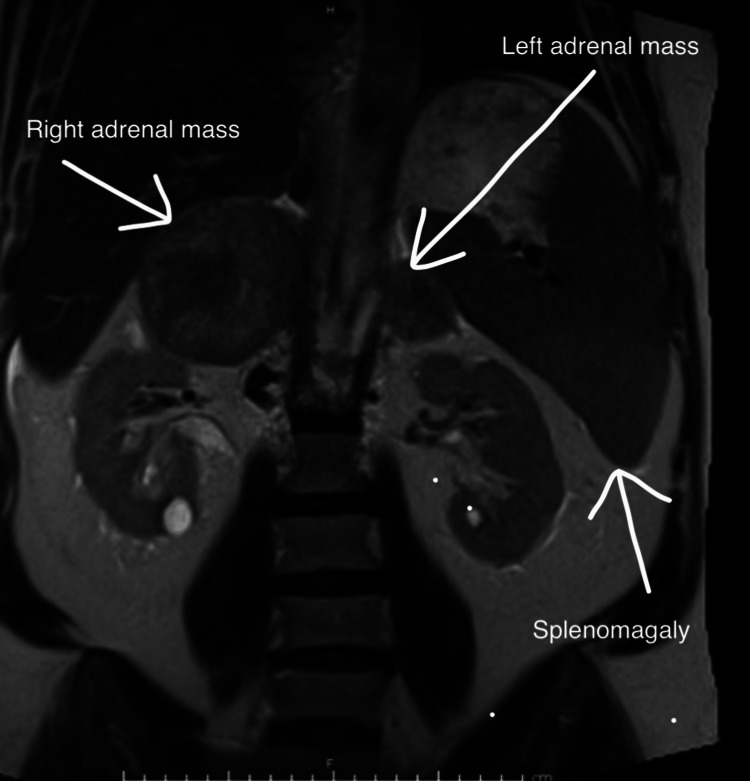
T2-weighted (T2W) MRI abdomen and pelvis Confirmed bilateral adrenal masses, with the right adrenal mass now measuring 8.5 cm. Splenomegaly was present. Features were suggestive of lymphoma versus metastases from an unknown primary. Multidisciplinary team (MDT) discussion was advised, and a biopsy was arranged and performed.

In August 2015, he re‑presented with anorexia, further weight loss, fever and left upper quadrant abdominal pain. Contrast‑enhanced CT of the abdomen and pelvis demonstrated bilateral adrenal masses (right 6.7 cm, left 4.5 cm) that were indistinct from the native adrenal glands, associated with splenomegaly (18 cm) and high non‑contrast attenuation (>20 Hounsfield units), in keeping with a malignant process (Figure 1). Adrenal MRI confirmed bilateral adrenal enlargement, with interval growth of the right mass to 8.5 cm and features suggestive of lymphoma or metastatic disease (Figure 2).

**Figure 3:**
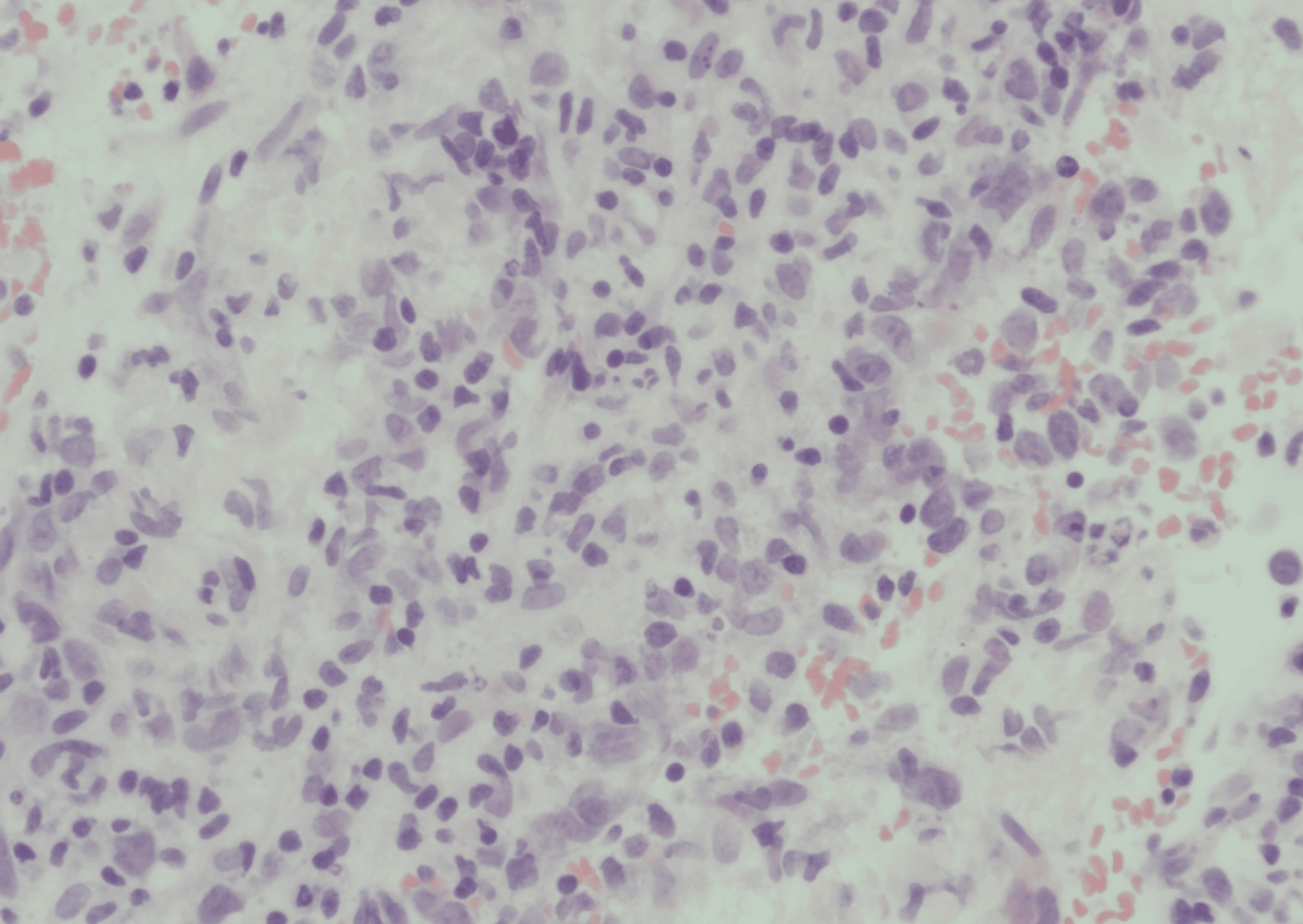
Histology picture of the needle core biopsy from the right adrenal gland 1 Cores of tissue consisting of a diffuse infiltrate of large lymphoid blasts with prominent nucleoli, smaller lymphoid cells and background inflammatory cells. Cores of tissue showing a diffuse population of blastic-type CD20+ lymphoid cells. The Ki-67 proliferation index is approximately 60%. The appearances are in keeping with a diffuse blastic B-cell lymphoma.

**Figure 4:**
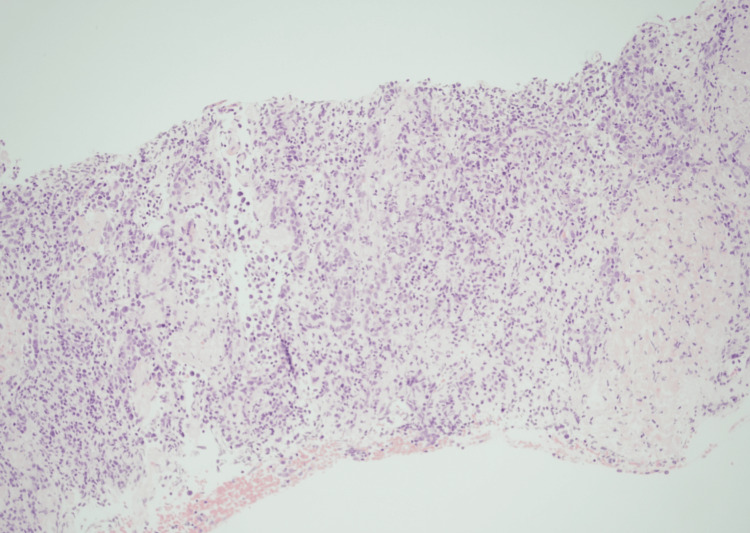
Histology picture of the needle core biopsy from the right adrenal gland 2 Cores of tissue consisting of a diffuse infiltrate of large lymphoid blasts with prominent nucleoli, smaller lymphoid cells and background inflammatory cells. Cores of tissue showing a diffuse population of blastic-type CD20+ lymphoid cells. Ki-67 proliferation index is approximately 60%. The appearances are in keeping with a diffuse blastic B-cell lymphoma.

Following a multidisciplinary review, an image-guided adrenal biopsy confirmed diffuse large B-cell lymphoma involving both adrenal glands (Figures 3-4), staged as stage IVB with bilateral adrenal involvement (Table 1). The case was reviewed by the All Wales Lymphoma Panel and the multidisciplinary team (MDT), and the final diagnosis was diffuse large B-cell lymphoma of the adrenal glands. In the absence of documented dominant nodal or other extra-adrenal disease, these findings support the diagnosis of primary bilateral adrenal lymphoma. Treatment was initially started urgently because he had severe abdominal pain, and the team felt they could not wait for final panel histology; one early cycle of rituximab, cyclophosphamide, doxorubicin (hydroxydaunomycin), vincristine (Oncovin) and prednisolone (R-CHOP) was delivered before the diagnosis pathway was fully completed, after which definitive treatment proceeded as R-CHOP.

**Table 1:** The reports of the needle core biopsy from the right adrenal gland EBER: Epstein-Barr virus-encoded small RNA; FISH: fluorescence in situ hybridisation; MYC: myelocytomatosis; LSI: locus-specific identifier

Needle core biopsy, right adrenal gland	Comments
Macroscopical report	Adrenal biopsy 18G core x 2 - 2 needle cores up to 8mm plus fragments.
Microscopical report	Cores of tissue showing a diffuse population of blastic-type CD20+ lymphoid cells. Ki-67 proliferation index is approximately 60%. The appearances are in keeping with a diffuse blastic B-cell lymphoma.
Histology	Cores of tissue consisting of a diffuse infiltrate of large lymphoid blasts with prominent nucleoli, smaller lymphoid cells and background inflammatory cells.
Immunohistochemistry	The blast-cells are CD20+, CD10-, BCL2+, BCL6+, MUM1+, EBER-. The small lymphocytes are CD3+. The Ki-67 index is 80%.
Immunostaining	There is no significant overexpression of MYC (30%). FISH using the Abbott LSI MYC Dual Colour Break Apart Probe (Abbott, Abbott Park, IL) showed no evidence of MYC gene rearrangement.
Conclusion	The appearances are of a diffuse large B-cell lymphoma with a non-germinal centre phenotype. Overall, there are no immunophenotypic or genetic adverse prognostic features other than non-germinal centre phenotype.

During the August 2015 admission, random cortisol was 124 nmol/L, which was inappropriately low in the context of acute illness, and the patient became hypotensive despite intravenous fluids, prompting treatment with hydrocortisone and fludrocortisone for suspected adrenal insufficiency. Short Synacthen testing in August 2015 showed a baseline cortisol of 220 nmol/L and a 30-minute cortisol of 353 nmol/L, which was suboptimal and supportive of adrenal insufficiency. Metadrenalines 24-hour urine test was within the normal range, showing no biochemical evidence of pheochromocytoma (Table 2).

The patient was regularly seen by the endocrinology team at the clinic after discharge. Follow-up testing confirmed persistent adrenal failure. Morning cortisol in February 2016 was less than 28 nmol/L, and repeat Short Synacthen testing in September 2016 showed a baseline cortisol of 41 nmol/L, rising only to 60 nmol/L at 30 minutes and 65 nmol/L at 60 minutes; this was a suboptimal response, and hydrocortisone replacement was continued. Adrenocorticotropic hormone levels measured in September 2016 were 147.9 ng/L (reference 7-63 ng/L), which strongly supported primary adrenal insufficiency due to bilateral adrenal involvement by lymphoma rather than central adrenal failure. Additional mineralocorticoid data were limited but helpful. Renin was elevated at 13.6 nmol/L/h in June 2017 (reference 0.5-3.5), supporting ongoing mineralocorticoid deficiency (Table 2). Fludrocortisone treatment was continued, with a later dose reduction from 0.1 mg to 0.05 mg daily, and then to alternate-day dosing when blood pressure became elevated. In August 2016, hydrocortisone was adjusted from 15 mg in the morning and 10 mg in the evening to 10 mg at 08:00, 5 mg at 12:00, and 5 mg at 16:00, while fludrocortisone was reduced from 100 micrograms daily to 50 micrograms daily due to weight gain and elevated blood pressure. Later, continued long-term glucocorticoid replacement, with hydrocortisone 10 mg /10 mg/5 mg in 2018-2021 and advice regarding sick-day rules and perioperative stress dosing, indicated persistent adrenal insufficiency without documented biochemical recovery. 

**Table 2:** Laboratory test results for endocrine work-up ACTH: adrenocorticotropic hormone; FSH: follicle-stimulating hormone; LH: luteinising hormone; TSH: thyroid-stimulating hormone; T4: thyroxine, or tetraiodothyronine

Parameters	August 2015	January 2016	February 2016	September 2016	November 2016	June 2017	October 2018	Reference
Random cortisol (nmol/L)	124	-	-	-	-	-	-	<450 does not exclude adrenal insufficiency
Morning cortisol (nmol/L)	-	-	<28	-	-	-	-	138 - 635
Short Synacthen test baseline morning cortisol (nmol/L)	220 nmol/L	-	-	41	-	-	-	-
30 minutes post 250ug Synacthen (nmol/L)	353	-	-	60	-	-	-	Cortisol normally reaches >450nmol/L 30 min post 250ug Synacthen
60 minutes post 250ug Synacthen (nmol/L)	-	-	-	65	-	-	-	-
Metadrenaline 24hr urine (µmol/24h)	0.29	-	-	-	-	-	-	-
Normetadrenaline 24hr urine (µmol/24h)	0.83	-	-	-	-	-	-	< 4.00
ACTH morning (ng/L)	-	-	-	147.9	-	-	-	7-63
Testosterone (nmol/L)	<0.5	-	37.8	3.0	1.0	14.6	5.7	9.7-38.2
FSH (IU/L)	2.9	-	-	3.2	5.4	-	5.4	1.0-12.0
LH (IU/L)	1.0	-	-	1.7	3.0	-	-	1.0-12.0
Prolactin (mU/L)	357	-	-	248	-	274	-	53-360
TSH (mU/L)	0.78	1.12	-	-	-	1.36	-	0.3-4.4
Free T4 (pmol/L)	13.0	15.3	-	-	-	13.9	-	9.0-19.1
Renin (nmol/L/h)	-	-	-	-	-	13.6	-	0.5-3.5

He also developed hypogonadism. In August 2015, testosterone was less than 0.5 nmol/L with low-normal gonadotropins (follicle-stimulating hormone, 2.9 IU/L and luteinizing hormone, 1.0 IU/L), while prolactin was within the reference range and thyroid function was normal, making primary testicular failure less likely on the documented data (Table 2). Testosterone remained abnormal on subsequent measurements, including 3.0 nmol/L in September 2016 and 1.0 nmol/L in November 2016, before improving with replacement therapy (Table 2). He was started by the endocrine team on intramuscular testosterone undecanoate during admission in August 2015, and later had treatment interrupted because of a rising PSA, then recommenced testosterone after urological assessment, including prostate MRI, showed no significant abnormality, and eventually transitioned to topical testosterone preparations, Testogel, during follow-up. No pituitary MRI was identified in the available records; accordingly, the hypogonadism is best described as biochemically hypogonadotropic, possibly functional in the context of severe systemic illness and chemotherapy. 

**Figure 5:**
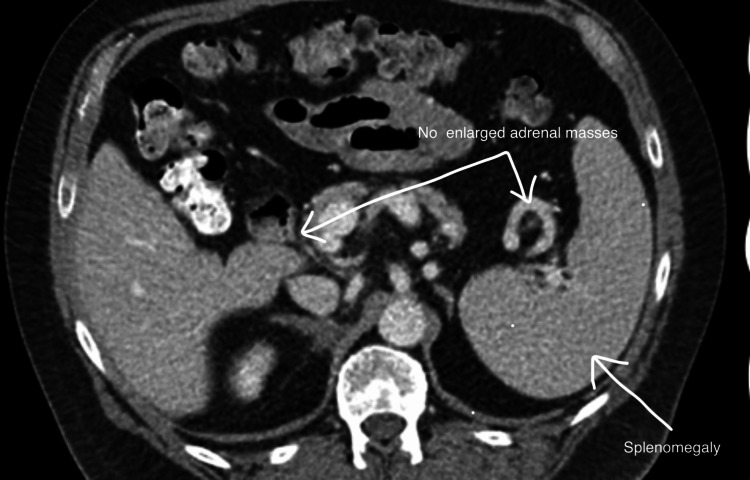
CT abdomen and pelvis post treatment No evidence of lymphoma relapse: There is no evidence of abnormal lymphadenopathy. Normal adrenals: The spleen is a little bulky but this has not changed.

**Figure 6:**
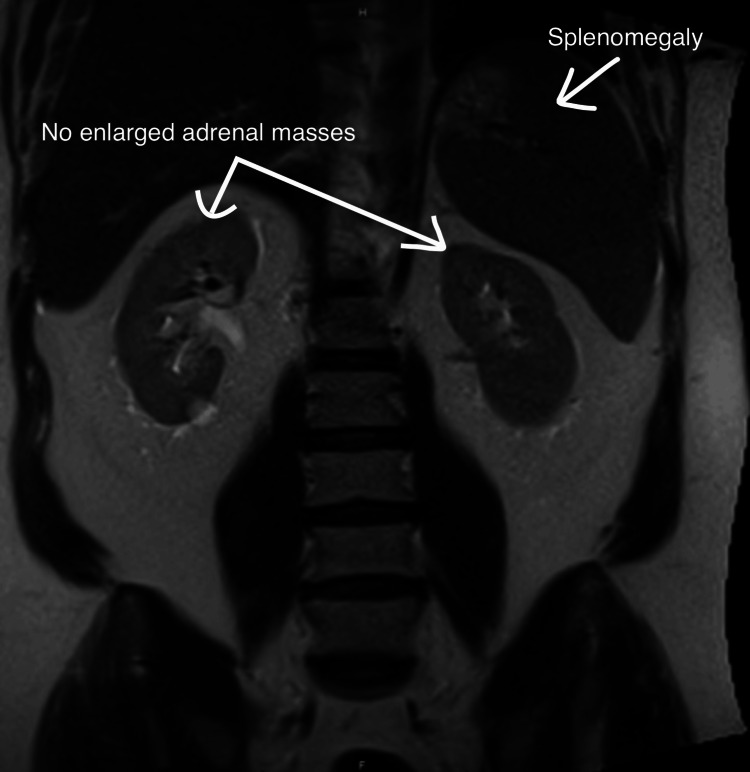
T2-weighted (T2W) MRI abdomen and pelvis post treatment No evidence of lymphoma relapse.

He received eight cycles of R-CHOP chemo-immunotherapy. Because adrenal involvement in diffuse large B-cell lymphoma is associated with a recognised risk of central nervous system (CNS) relapse, CNS prophylaxis was also planned, and in January 2016, prophylactic intrathecal methotrexate 12.5 mg was administered under radiological guidance. The patient received two doses of intrathecal prophylactic methotrexate. During treatment, he developed probable *Escherichia coli* meningitis with persistent headaches after lumbar punctures, and in February 2016, because of these CNS symptoms and possible meningitis, the haematology team decided no further intrathecal prophylaxis would be given. CNS prophylaxis was chosen because of the high-risk adrenal site, but was curtailed after two doses because of infection and procedure-related complications. Despite incomplete CNS prophylaxis, the overall lymphoma response was excellent. End-of-treatment CT and MRI showed the disappearance of the prior lymphadenopathy and the return of the adrenal glands to normal size (Figures 5-6). PET imaging in 2016 demonstrated complete metabolic remission. Subsequent haematology follow-up through 2020 continued to describe ongoing complete remission without clinical evidence of relapse. There is no documented evidence in the available records of CNS relapse.

In 2023, CT was performed because of abdominal symptoms, raising concern for recurrence. Still, MRI of the liver and associated correspondence concluded that the dominant process was decompensated cirrhosis without evidence of hepatocellular carcinoma, and the adrenal glands were described as unremarkable on imaging. The patient later died in 2023 from sepsis and multi-organ failure, with no documented evidence of lymphoma relapse before death. 

## Discussion

This case highlights several characteristic but easily missed features of PAL: constitutional symptoms, large bilateral adrenal masses, adrenal insufficiency, and rapid progression in the absence of early diagnosis. PAL typically affects men in the sixth to seventh decade and often presents with B‑symptoms, abdominal discomfort, or adrenal crisis; bilateral disease and adrenal insufficiency are strongly associated, although adrenal failure can also occur with unilateral involvement [4,5].

Imaging plays a key role in raising suspicion for PAL; in the largest series of 50 patients, the median non‑contrast CT attenuation was about 32 Hounsfield units, with no lesions measuring below 10 Hounsfield units and most showing heterogeneous, ill-defined margins [2,8,9]. However, high attenuation and low washout are not specific for malignancy, as lipid-poor adenomas, pheochromocytomas, infections, and other infiltrative processes can appear similar; therefore, lesions with attenuation greater than 20 Hounsfield units are best regarded as indeterminate or suspicious for non-adenomatous disease rather than diagnostic of malignancy alone [6,10,11]. In this patient, the combination of bilateral high‑attenuation adrenal masses with splenomegaly, constitutional symptoms and evolving adrenal insufficiency made lymphoma particularly likely and justified early biopsy.

The differential diagnosis of large bilateral adrenal masses should therefore remain broad. Important considerations include metastatic disease, adrenocortical carcinoma, infection such as tuberculosis, haemorrhage, lymphoma and pheochromocytoma. Several case series and reviews emphasise that PAL is often misdiagnosed as metastatic carcinoma, and that early CT-guided adrenal biopsy is critical to secure the diagnosis and avoid delay in initiating therapy [3,4,12,13].

Adrenal insufficiency occurs in about two‑thirds of bilateral PAL cases and carries important prognostic and therapeutic implications. Rashidi and Fisher’s systematic review of 187 cases found that adrenal insufficiency did not correlate directly with tumour size and could occur even in unilateral disease, suggesting that lymphoma‑related destruction or infiltration of adrenal cortex, vascular compromise or cytokine‑mediated dysfunction may contribute [1]. Early recognition and immediate glucocorticoid replacement are essential, as adrenal crisis is a major cause of morbidity and mortality, and steroid therapy should not be delayed while awaiting confirmatory testing when primary adrenal insufficiency is suspected [4,8,9,14,15].

Regarding treatment, combination chemo‑immunotherapy with R‑CHOP remains the mainstay, often combined with CNS prophylaxis due to the reported risk of CNS involvement. Although the overall prognosis is poor, with a median survival of approximately 14 months in some cohorts, long‑term remission has been reported, particularly when the diagnosis is made early and adequate steroid replacement and full‑dose R‑CHOP can be delivered. This patient’s durable remission from 2016 to 2023 suggests that earlier recognition of adrenal insufficiency and timely chemo‑immunotherapy can yield favourable long‑term outcomes despite the generally aggressive nature of PAL [2-4,8,9,11,12].

Clinically, this case reinforces several important learning points: PAL should be considered in patients with bilateral adrenal masses, particularly when these are accompanied by B‑symptoms or biochemical evidence of adrenal failure. High‑attenuation adrenal lesions with low washout and large size should raise suspicion for adrenal lymphoma and lead to early biopsy in a multidisciplinary setting. In addition, prompt institution of hydrocortisone at presentation and continuation throughout chemotherapy is critical to prevent adrenal crisis and to optimise tolerance of systemic therapy.

## Conclusions

This case describes a 50‑year‑old man whose initially non‑specific constitutional symptoms evolved into adrenal insufficiency and led to the diagnosis of primary bilateral adrenal lymphoma presenting as rapidly enlarging, high‑attenuation adrenal masses with splenomegaly confirmed as diffuse large B‑cell lymphoma on biopsy. He required long‑term hydrocortisone and fludrocortisone replacement and received eight cycles of R‑CHOP with intrathecal methotrexate, achieving complete radiological remission that was sustained for seven years until his death from sepsis without evidence of lymphoma relapse. The case highlights that bilateral adrenal enlargement with B‑symptoms and biochemical adrenal failure should raise prompt suspicion for PAL, that large, high‑attenuation adrenal lesions with low washout merit early multidisciplinary review and CT‑guided biopsy, and that immediate and continued glucocorticoid replacement alongside full‑dose chemo‑immunotherapy is crucial both to prevent adrenal crisis and to allow delivery of effective systemic treatment.

This case highlights that bilateral adrenal enlargement accompanied by B-symptoms and biochemical adrenal insufficiency should prompt consideration of PAL. Early multidisciplinary evaluation with CT-guided biopsy, together with timely glucocorticoid replacement and chemo-immunotherapy, is essential to prevent adrenal crisis and optimize outcomes.
